# Patterns of Coupled Theta Activity in Amygdala-Hippocampal-Prefrontal Cortical Circuits during Fear Extinction

**DOI:** 10.1371/journal.pone.0021714

**Published:** 2011-06-28

**Authors:** Jörg Lesting, Rajeevan T. Narayanan, Christian Kluge, Susan Sangha, Thomas Seidenbecher, Hans-Christian Pape

**Affiliations:** 1 Institute of Physiology 1, Westfälische-Wilhelms-Universität, Münster, Germany; 2 Department of Neurology, Otto-von-Guericke-Universität, Magdeburg, Germany; 3 Institute of Cognitive Neuroscience, University College London, London, United Kingdom; 4 Wellcome Trust Centre for Neuroimaging, University College London, London, United Kingdom; 5 Institute for Experimental Epilepsy Research, Westfälische-Wilhelms-Universität, Münster, Germany; University of Wuerzburg, Germany

## Abstract

Signals related to fear memory and extinction are processed within brain pathways involving the lateral amygdala (LA) for formation of aversive stimulus associations, the CA1 area of the hippocampus for context-dependent modulation of these associations, and the infralimbic region of the medial prefrontal cortex (mPFC) for extinction processes. While many studies have addressed the contribution of each of these modules individually, little is known about their interactions and how they function as an integrated system. Here we show, by combining multiple site local field potential (LFP) and unit recordings in freely behaving mice in a fear conditioning paradigm, that theta oscillations may provide a means for temporally and functionally connecting these modules. Theta oscillations occurred with high specificity in the CA1-LA-mPFC network. Theta coupling increased between all areas during retrieval of conditioned fear, and declined during extinction learning. During extinction recall, theta coupling partly rebounded in LA-mPFC and CA1-mPFC, and remained at a low level in CA1-LA. Interfering with theta coupling through local electrical microstimulation in CA1-LA affected conditioned fear and extinction recall depending on theta phase. These results support the hypothesis that theta coupling provides a means for inter-areal coordination in conditioned behavioral responsiveness. More specifically, theta oscillations seem to contribute to a population code indicating conditioned stimuli during recall of fear memory before and after extinction.

## Introduction

Control of fear and anxiety relies on a neural system, which can be experimentally assessed through Pavlovian fear conditioning [1]. In this paradigm, a subject learns to associate an initially neutral conditioned stimulus (CS) with a coinciding aversive unconditioned stimulus (US), such that subsequent exposure to the CS elicits conditioned fear responses. Repeatedly presenting the CS alone attenuates the fear response, a process known as fear extinction [2]. The mechanisms underlying fear extinction have attracted considerable interest because of their potential clinical significance (for review see [3], [4]). Convergent evidence indicates that interconnections between amygdala, hippocampus and medial prefrontal cortex (mPFC) are crucial for the physiological regulation of fear memory formation and extinction [2], [3], [5], [6]. Collectively, the current literature [2], [3], [5], [6] suggests that underlying synaptic circuits involve the lateral amygdala (LA) for forming and maintaining CS-US associations, the hippocampus for context-dependent modulation, and the infralimbic medial prefrontal cortex (mPFC) for extinction-related processes. While many studies have addressed the contribution of each of these modules to fear learning and memory individually, comparatively little is known about their interactions and how they function as an integrated system. A candidate mechanism for reliable yet flexible integration of functions within this tripartite circuit is synchronization in the theta frequency range. As is widely accepted, such oscillations can yield spatiotemporal codes [7], and provide a means for temporal compression from the rather long time scale of learned behavior down to the millisecond timescale required for synaptic plasticity [8]–[11].

While the hippocampus is central in the physiology of theta oscillations [7], [12], theta activity has also been observed in the basolateral complex of the amygdala, for instance during paradoxical sleep [13] and during the anticipation of noxious stimuli [14]. Within the mPFC, neurons are modulated by hippocampal theta [15], [16], and an increase in theta power occurs during acquisition of trace-conditioning [17]. Furthermore, there is evidence for conditioned fear-related theta activity in the mPFC [18], and hippocampal-mPFC theta coupling increases with anxiety [19]. Theta coupling is high between the hippocampal subfield CA1 and the LA during consolidation [20] and reconsolidation [21] of conditioned fear, and decreases at remote stages [22]. In keeping with this, REM-associated theta coupling in BLA, hippocampus and mPFC was found to relate to consolidation success in fear conditioning [23].

Theta activity thus seems to constitute an integrative mechanism for coordination of activity in the fear memory network. While recent findings have highlighted the functional impact of theta coupling in amygdala-hippocampal-prefrontal cortical circuits [23], a systematic study on theta specificity for fear extinction is missing. Using multiple site recordings of local field potentials (LFPs) and unit activity in freely behaving mice, as well as electrical microstimulation techniques, we show here that theta cross-correlations closely resemble the behavioral dynamics across recall of fear memory and extinction.

## Results

**Figure 1:**
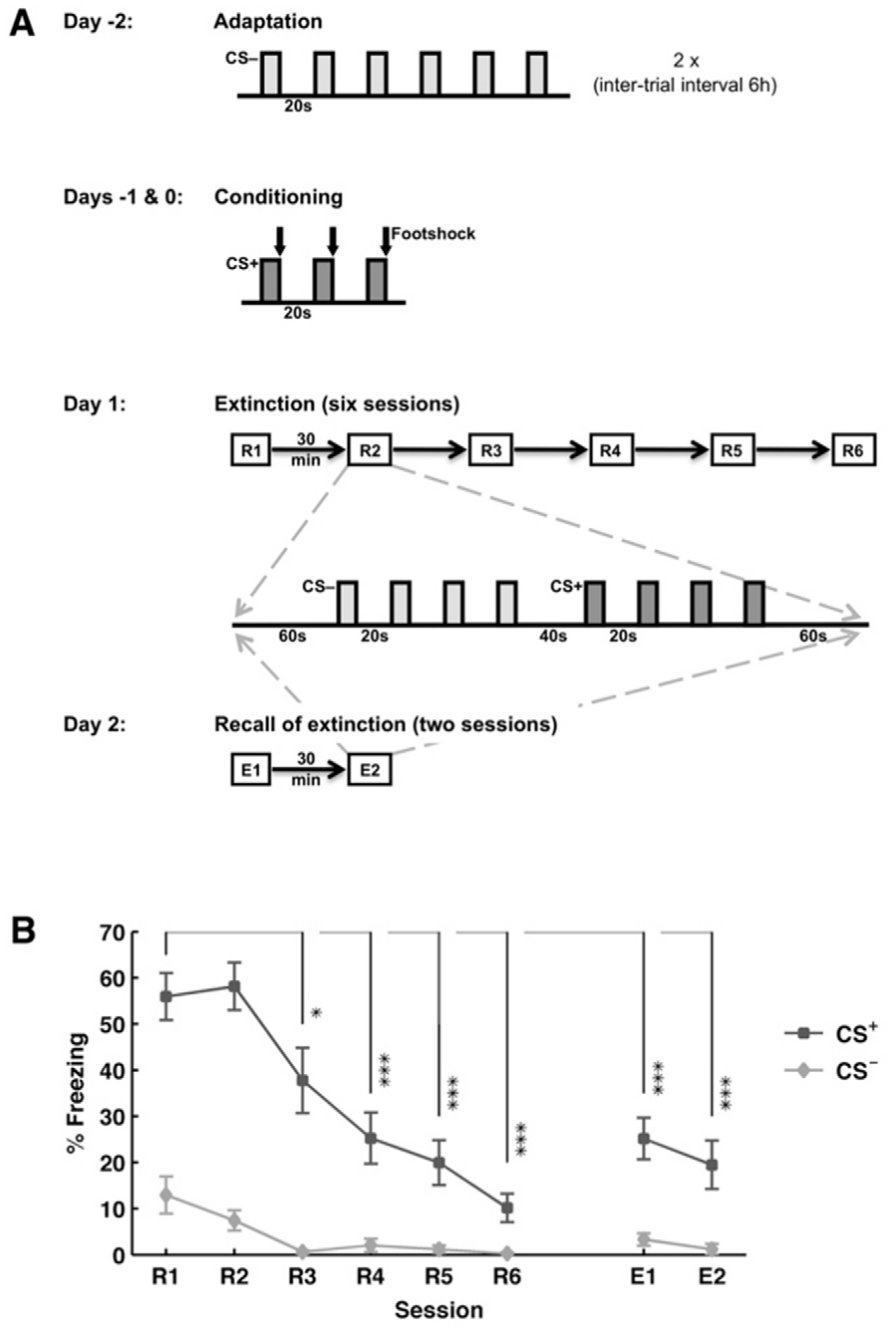
Experimental Design (A). During adaptation animals (n = 13) were exposed to six CS- only and the entire session was repeated six hours later. Conditioning took place on the following two days (days -1 and 0): The CS+ was presented three times during each conditioning session, every time co-terminating with an electric footshock. Memory was tested on days 1 and 2. On day 1, six consecutive retrieval sessions were carried out (R1 through R6), with 30 minutes between sessions. Extinction memory was recalled on day 2 in two sessions (E1 and E2), again separated by 30 minutes. All sessions on days 1 and 2 were identical (see inset), and contained four CS- and four CS+ presentations. **CS-evoked conditioned freezing (B).** The fraction of time spent freezing during presentation of the CS+ declined over extinction sessions (R1-6) and remained low during extinction memory recall (E1&2). Note the very low levels of conditioned freezing during CS- presentations. Freezing in response to the CS+ was compared across sessions, revealing that, from session R3 onwards, the observed freezing values significantly differed from R1. Values are mean ± SEM; asterisks indicate the significance level: *, p<0.05; **, p<0.01; ***, p<0.001.

Mice were fear-conditioned using an auditory Pavlovian paradigm (Figure 1A). Conditioned fear behavior was assessed during successive retrieval sessions (R1-R6), with repetitive presentations of conditioned (CS+) and neutral stimuli (CS-) for fear extinction, and during recall of fear extinction (E1, E2) twenty-four hours later. Behaviorally, animals (n = 13) displayed a high degree of freezing upon CS+ presentation in early retrieval sessions, which decreased over the course of extinction (Figure 1B). One-way ANOVA revealed a significant effect of session on CS+ - elicited freezing (F_6,96_ = 10.12, p<0.000000001), and post-hoc multiple comparison (Bonferroni) showed that, from session R3 onwards, freezing values differed significantly from R1 (R1 (55.9±5.1%); R3 (37.8±7.0%): p<0.05; R4 (25.2±5.6%): p<0.0001; R5 (20.0±4.9%): p<0.00001; R6 (10.1±3.1%): p<0.000001; E1 (25.2±4.5%): p<0.0001; E2 (19.48±5.3%): p<0.00001). In addition, the amount of freezing in response to the CS+ was significantly greater than that to CS- (t-test, p<0.01) in all sessions.

### Theta cross-correlation during retrieval of conditioned fear and fear extinction

**Figure 2:**
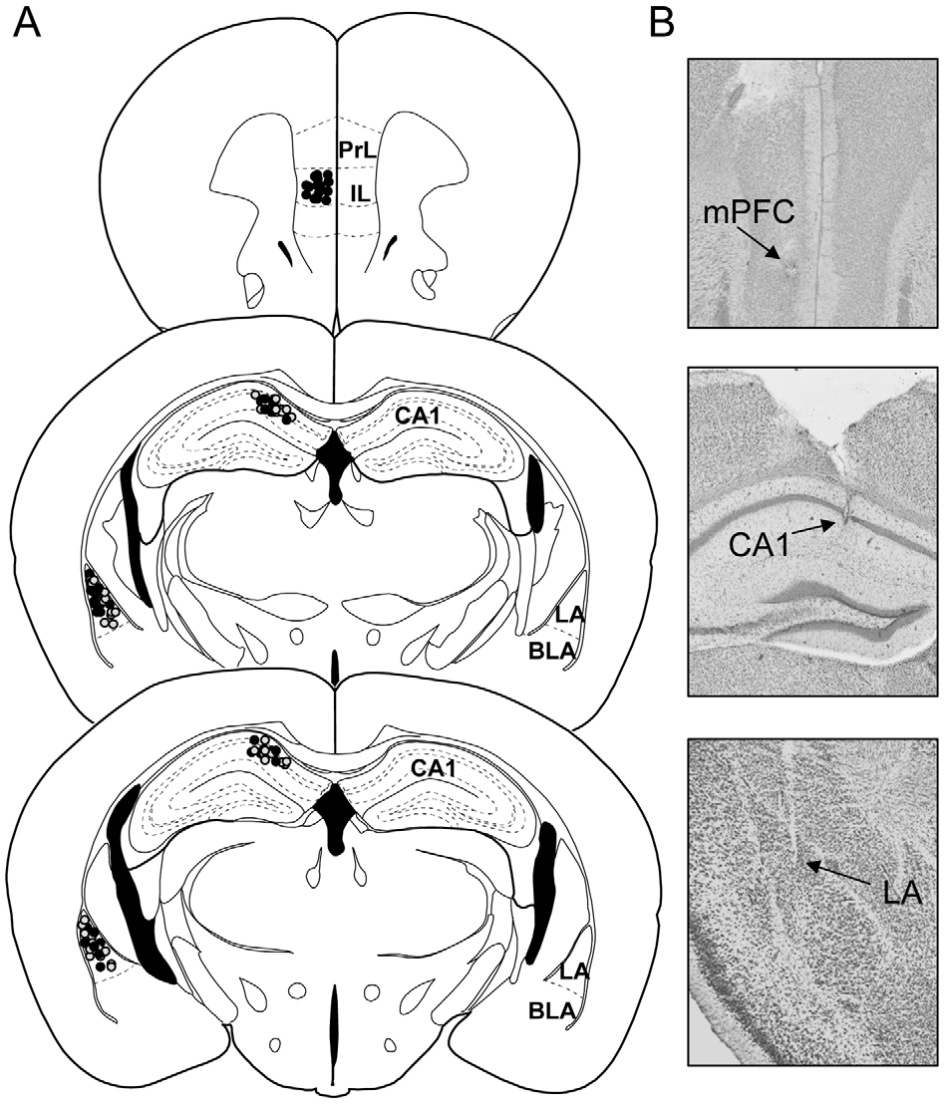
Verification of recording sites. (A) Schematic representation of electrode locations in the medial prefrontal cortex (mPFC), hippocampal CA1, and lateral amygdala (LA). Black dots mark field potential and unit activity recordings sites; grey dots represent sites of electrical microstimulation. (B) Representative Nissl stained coronal sections showing electrode positions in the mPFC, CA1 and LA. Arrows indicate the electrode tip positions. PrL, prelimbic region of mPFC; IL, infralimbic region of mPFC; BLA, basolateral amygdala.

**Figure 3:**
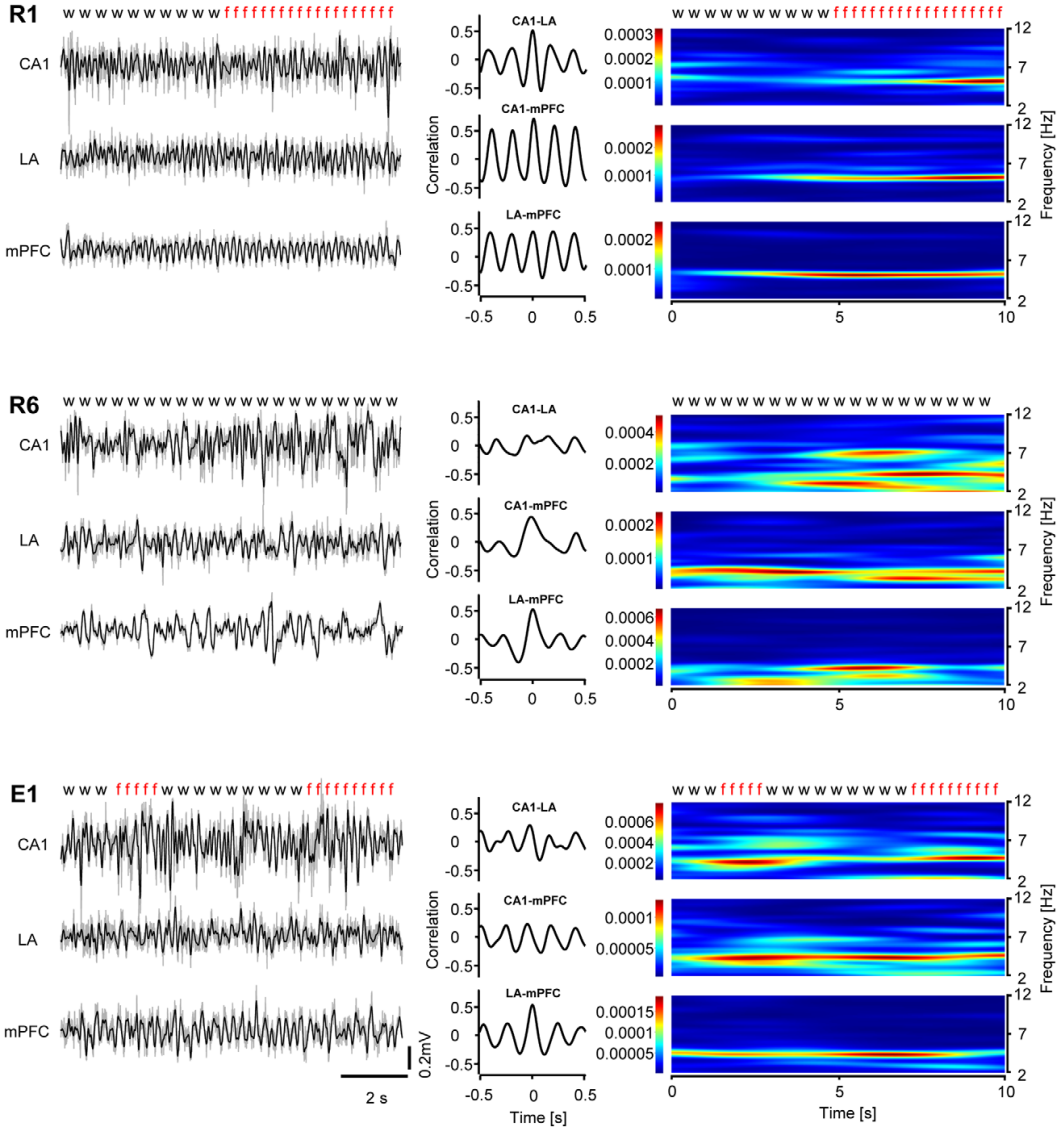
Representative data from one animal in all three channels (CA1, LA, mPFC) during CS+ presentation at R1 (top), R6 (middle) and E1 (bottom). For each session, LFP waveforms (left) as raw (light colored) and 2–12 Hz filtered (dark colored) traces, cross-correlograms (middle) and time-frequency representations (right) are shown. Note the coupled theta activity, evident as periodic patterns in the cross-correlograms, among all three channels during R1. This effect is absent at R6, while at E1 synchronous activity emerges again, predominantly in the CA1-mPFC and the mPFC-LA pathways. Characters indicate animal behavior (f: freezing; w: risk-assessment).

**Figure 4:**
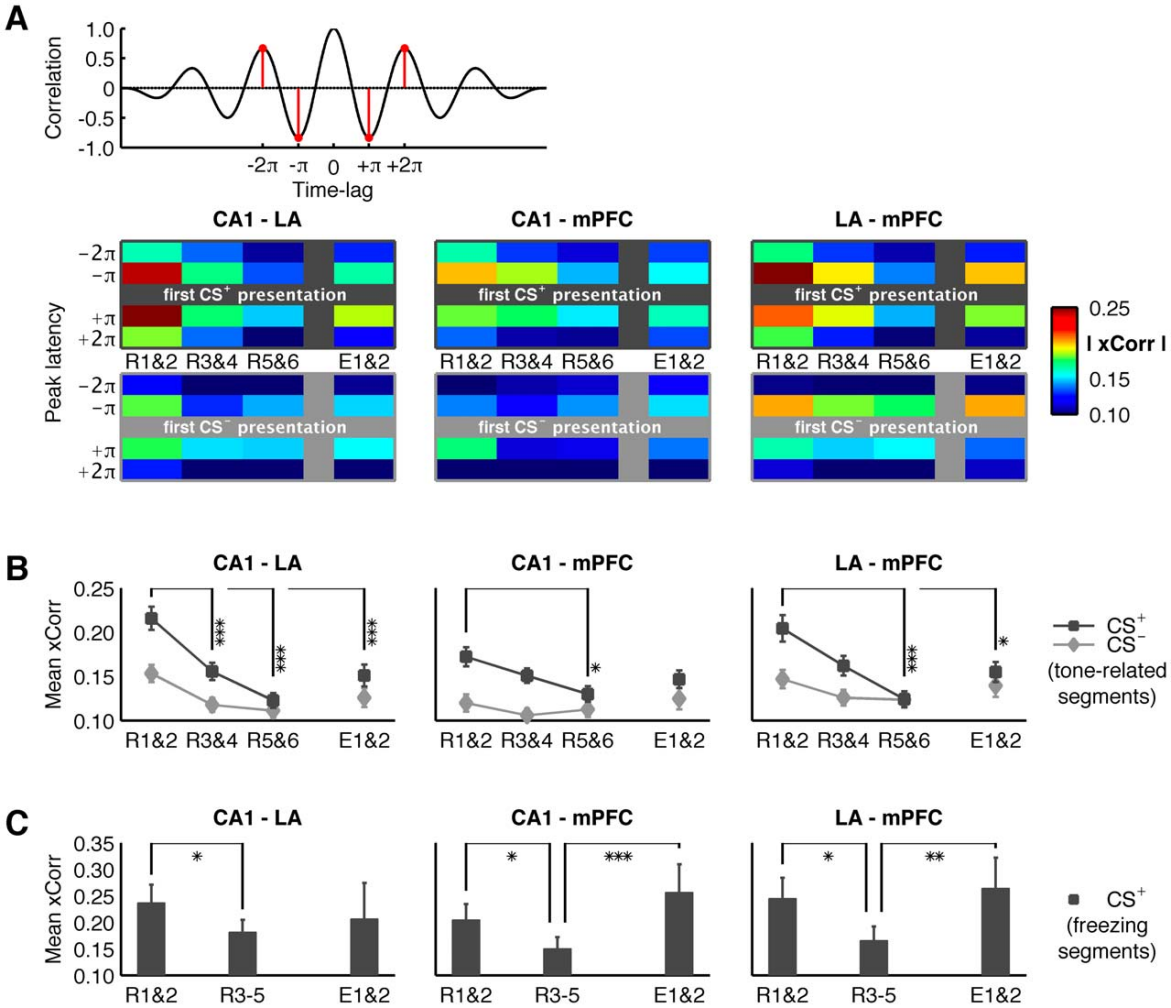
Group averages from recordings in 13 animals of cross-correlation between LA, CA1 and mPFC during extinction (R1-R6) and extinction recall (E1 & E2). (A) Magnitude of cross-correlation waveforms at different latencies (±π, ±2π) during presentation of CS+ (top) and CS- (bottom). Note the decrease in cross-correlation magnitude evident in all channel combinations as extinction progresses. (B) Mean cross-correlation values (averages of ±π, ±2π), summing up the panels presented in (A). Coupling clearly decreases over the course of extinction in all channel pairs. (C) Mean cross-correlation values as shown in (B), however, considering only periods during which the animal actually displayed freezing while exposed to the CS+. Sessions R3-5 are binned, due to the little freezing observed from R3 onwards (no freezing periods survived artifact rejection for R6). Likewise, freezing was rarely observed in response to CS- and is therefore not quantified. Note the similar dynamics over sessions in comparison to (B). Values are mean ± SEM. in all plots; asterisks indicate the significance level: *, p<0.05; **, p<0.01; ***, p<0.001.

LFP recordings were obtained simultaneously from CA1, LA and the infralimbic region of the mPFC in a total of 13 mice. LFP were combined with extracellular unit recordings in these regions in a separate group of animals (n = 8). Only data from histologically verified recording sites were included in the analysis (Figure 2). Across animals, theta activity was consistently observed in the LFPs recorded at CA1, LA, and mPFC upon CS+ and CS- presentation (Figure S1), and theta phase-related unit activity occurred in all regions of interest (Figure S2), indicating the robustness of theta and contribution of local network processes under the given experimental conditions. Cross-correlation analyses indicated that theta activity was highly correlated between regions during early retrieval sessions (R1&2) and during recall of extinction (E1&2), while correlation was weak or absent during late retrieval sessions (R3-R6). An example is illustrated in Figure 3. In all recorded animals (n = 13) theta activity was systematically analyzed via cross-correlation series between all possible pairs of regions (CA1-LA, CA1-mPFC, LA-mPFC), and the magnitudes at half a cycle (±π) and one full cycle (±2π) were taken as measures for oscillatory interactions (Figure 4A). The first two retrieval sessions (R1&R2) were characterized by a substantial linear relation (high cross-correlation magnitudes) in all three pairs of regions (Figure 4A, B). Correlation declined during subsequent retrieval sessions (R3&R4) between all three regions and was no longer observed at later sessions (R5&R6). Importantly, correlations remained at low levels upon CS- presentation throughout the entire experiment (Figure 4A, B). Statistical assessment through ANOVA (F_5,150_ = 8.86, p<0.000001) revealed significant effects of session (F_3,150_ = 18.50, p<0.0001) and stimulus (F_1,150_ = 35.56, p<0.0001), as well as a significant interaction (F_5,150_ = 7.79, p<0.0001). Similarly, the averages of the four peak values (±π, ±2π), taken as gross measures for entrainment in a two cycle time window, showed a significant effect of session (separate one-way ANOVAs for each channel pair considering CS+ only, p<0.0001 for all pairs). Specifically, as revealed by post-hoc assessment (Bonferroni), in CA1-LA, all subsequent sessions showed less entrainment than R1&2 (p<0.0001 for all other sessions; correlation values: R1&2: 0.22±0.01; R3&4: 0.16±0.01; R5&6: 0.12±0.01; E1&2: 0.15±0.01). In CA1-mPFC, R3&4 showed a strong trend and R5&6 proved significantly lower than R1&2 (p<0.05; correlation values: R1&2: 0.17±0.1; R3&4: 0.15±0.1; R5&6: 0.13±0.1), while E1&2 did not differ from the initial retrieval sessions (E1&2: 0.15±0.1). In LA-mPFC, the pattern appeared similar to CA1-LA: From R5&6 onwards, a significant decrease of entrainment was observed (p<0.001 for R5? p<0.05 for E1? correlation values: R1&2: 0.20±0.1; R3&4: 0.16±0.1; R5&6: 0.12±0.1; E1&2: 0.15±0.1).

Next, as increases in theta coupling in CA1-LA have previously been related to conditioned fear behavior [22], [23], analyses were confined to data segments during which the animals displayed freezing while exposed to the CS+. Due to low freezing levels at late retrieval sessions (see Figure 1B), data were pooled from sessions R1&2, R3-5, and E1&2 to assure appropriate sample sizes (data segments: R1&2: n = 26; R3-5: n = 24; E1&2: n = 11). For all channel pairs one-way ANOVA revealed significant changes over the sessions (CA1-LA: F_2,58_ = 3.98, p<0.05; CA1-mPFC: F_2,58_ = 9.21, p<0.001; LA-mPFC: F_2,58_ = 7.26, p<0.01). Bonferroni post-hoc analysis showed a significant decrease in theta cross-correlation between all areas during repeated retrieval sessions (R1&2 versus R3-R5) (CA1-LA: p<0.05; R1&2: 0.23±0.1; R3-5: 0.17±0.1; E1&2: 0.21±0.3/CA1-mPFC: p<0.05; R1&2: 0.20±0.2; R3-5: 0.15±0.1; E1&2: 0.26±0.3/LA-mPFC: p<0.01; R1&2: 0.25±0.2; R3-5: 0.17±0.1; E1&2: 0.26±0.3; see Figure 4C). During recall of extinction (E1&2), theta coupling significantly increased in CA1-mPFC (p<0.001 compared to R3-R5) and LA-mPFC (p<0.01 compared to R3-R5) (Figure 4C). By comparison, in CA1-LA, theta coupling remained at a low level during recall of extinction similar to that during repeated retrieval sessions (p = 0.72 E1&2 compared to R3-R5; Figure 4C). Of note, theta cross-correlations were not significantly different at E1&2 compared with R1&R2 between any of the areas tested.

### Theta Burst Stimulation of CA1 and LA

**Figure 5:**
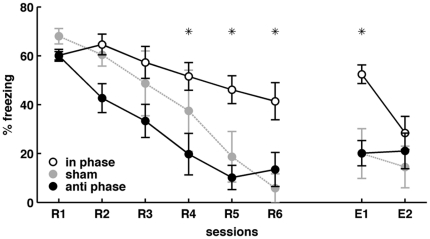
Effects of theta burst stimulation on CS+ - evoked freezing. Trains of stimuli were delivered locally to CA1 and LA at the same phase (“in phase”; n = 7 animals tested) or at 180° phase offset (“anti phase”; n = 8), after each CS+ presentation in each retrieval session (R1-R6) during extinction learning. Note the delay in extinction of conditioned freezing and maintained fear responsiveness during extinction recall in the “in phase” group (n = 7) as compared to “sham” controls (n = 6). Asterisks indicate significant differences “in phase” vs. “anti phase” (p<0.05). Values are mean ± SEM.

In order to experimentally assess the possible influence of theta coupling on freezing behavior, patterned theta stimuli were simultaneously delivered to the LA and CA1 via electrical microstimulation. The rational for interfering with theta coupling at the CA1-LA connection was as follows: when considering freezing periods (see Figure 4C), theta coupling between these two areas significantly decreases during extinction learning, and remains at low levels during extinction recall, thereby differing from the other two channel pairs which showed a significant theta coupling rebound. Therefore, these two sites proved particularly suited for probing the behavioral consequences of electrical theta stimulation as an intervention testing the predictions of our coupling analyses. Stimuli were applied following CS+ presentation in each retrieval session, and CA1 and LA were stimulated either synchronously (“in phase”), or with a phase offset of 180° between sites (“anti phase”). In a period following “in phase” theta stimulation, an increase in correlated theta activity was observed in CA1 and LA (Figure S3 A, B). This was a transient phenomenon, in that it did not persist during sessions with no concomitant stimulation on the following day (Figure S3 A, B). Furthermore, theta stimulation was found to not induce aversive behavior in animals (n = 5) that had not been fear-conditoned but otherwise went through the routine sessions of CS presentations (Figure S3 C). In the group of fear-conditoned animals, “in phase” theta burst stimulation was associated with a delay in extinction of conditioned freezing and maintained fear responsiveness during extinction recall (n = 7), as compared to non-stimulated “sham” controls (n = 6; Figure 5). “Anti phase” stimulation probed in a separate group of conditioned animals (n = 8), resulted in a rapid decrease in fear responsiveness during extinction learning and unchanged extinction recall, as compared to non-stimulated “sham” controls (n = 6; Figure 5). One-way repeated measures ANOVA reavealed main effects of group (F_2,18_ = 7.58, p<0.01), session (R1-E2; F_7,126_ = 20.57, p<0.001) as well as an interaction between groups and session (F_14,126_ = 2.14, p<0.05; Figure 5). Between groups the following differences emerged (Bonferroni post-hoc test): “sham” versus “in phase”, R6-E1, p<0.05 (sham R6 (5.8±5.8%), E1 (20±10.16%); in phase R6 (41.4±7.6%), E1 (52.4±3.8%)), and “in phase” versus “anti phase”, R4-E1, p<0.05 (in phase R4 (51.6±5.7%), R5 (46.1±5.7%); anti phase R4 (19.8±8.5%), R5 (10.1±4.9%), R6 (13.4±7%), E1 (20.1±5.2%)). No significant differences were observed between the “sham” and “anti phase” groups. Within-group differences across extinction training and recall of extinction were analyzed via one-way ANOVA followed by post-hoc multi comparison testing (Bonferroni). The “sham” group showed a reduction of freezing behavior over extinction training (R1-R6) which remained low during E1, demonstrating successful extinction of fear (F_7,40_ = 5.26, p<0.001; post hoc: R5-E2, p<0.05, compared to R1; R1 (68±3.1%), R5 (18.7±10.3%), R6 (5.8±5.8%), E1 (20±10.16%), E2 (14.5±8.5%)); freezing levels were significantly reduced from R1 beginning at R5. Furthermore, performing one-way repeated measures ANOVA comparing animals from LFP recordings (Figure 1B) and “sham” controls, revealed no significant differences between the groups. “Anti phase” stimulated animals showed a significant reduction in freezing levels from R3 onwards (F_7,56_ = 7.31, p<0.001; post hoc: R4-E2, p<0.01 compared to R1; R1 (59.9±1.7%), R3 (33.3±6.8%), R4 (19.8±8.5%) R5 (10.2±4.9%), R6 (13.4±6.9%), E1 (20.1±5.2%), E2 (21.1±7.3%)). Conversely, the “in phase” stimulated animals expressed delayed fear extinction with no significant reduction in freezing until E2. Only at E2 freezing levels were significantly lower, when compared to R1 (F_7,48_ = 4.27, p<0.001; post hoc: E2, p<0.01; R1 (60.18±2.4%), E2 (28.4±6.8%)).

## Discussion

Our findings indicate that aversive responses to fear-conditioned stimuli during distinct phases of fear retrieval, extinction learning and extinction recall are associated with theta coupling in a synaptic network comprising LA, CA1 and the infralimbic region of mPFC. Theta cross-correlations increase during retrieval of fear memory, decline during extinction learning and partly rebound during extinction recall in a regionally specific manner. Furthermore, artificial theta entrainment of CA1 and LA through microstimulation leads to maintained conditioned fear responses during extinction. In the following, we discuss how theta coupling may organize functional modules within these synaptic networks, providing a neurophysiological mechanism for functional coordination of remote areas during stages of memory retrieval and extinction.

Theta interactions have emerged as candidates for organizing activity in synaptic pathways of conditioned fear. In particular, coupled theta activity increases in CA1-LA during CS+ - evoked freezing at long-term but not short-term memory stages [20]–[22], [24], [25], REM-associated theta coupling in BLA, hippocampus and mPFC is related to consolidation success in fear conditioning [23], and theta increases in mPFC during fear extinction [18]. Furthermore hippocampal-mPFC theta coupling increases with anxiety [19]. The present study significantly extends these findings by demonstrating that theta within CA1, LA, and mPFC is organized in a regionally specific manner during conditioned fear responses at stages of memory retrieval and extinction. More specifically, theta entrainment involved CA1, LA and mPFC during memory retrieval, and theta coupling overall declined during extinction learning. CS+ - evoked freezing during extinction recall was accompanied by a partial rebound of theta coupling between mPFC-CA1 and mPFC-LA, leaving CA1-LA coupling on a relatively low level. Importantly, theta coupling in these areas is not a mere reflection of the level of fear expression, but related to the success of fear conditioning, suggesting involvement in fear memory consolidation [22], [23]. In keeping with this, short trains of theta frequency stimulation applied to CA1 and LA during extinction learning, meant to mimic LA-CA1 coupling typical of fear memory consolidation, resulted in prolonged CS+ - induced freezing during extinction learning and impaired extinction recall. This effect was observed when theta stimuli were applied “in phase” (but not with a phase shift of 180°), which did not evoke aversive behavior itself (Figure S3). In fact, the employed type of short synchronous stimulation has previously been used to enhance the excitability of CA1 neurons in an input specific and prolonged manner [26]. A phase shift of 180° was then chosen as disruptive protocol because it would bring about the inverse of the high entrainment pattern observed during early retrieval, where the central cross-correlation peaks occur at very short latencies, indicative of nearly peak-to-peak coupling. In the “anti phase” group, CS+ induced freezing was readily reduced during extinction learning and remained at low levels, indistinguishable from controls during recall of extinction at E1 and E2. Of note, theta stimulation was carried out without a specific relationship to the ongoing physiological theta in both regions. Coupled theta activity in CA1-LA (and mPFC) has been shown to determine the success of conditioned fear, most likely through an effect on memory consolidation [22], [23]. The application of theta stimuli is thus not likely to induce fear learning in non-trained animals (Figure S3), but to modulate the conditioning success after fear training (Figure 5). The obtained data are in line with these hypotheses, and overall corroborate the notion that high degrees of theta coupling between CA1, LA and PFC promote conditioned fear responses [23].

The present results, in addition, indicate that a decrease in theta coupling in LA, CA1 and mPFC characterizes extinction learning, and that conditioned fear responses during extinction retrieval are associated with a partial rebound of theta coupling, mostly involving mPFC-LA and mPFC-CA1 interactions. Whether or not these changes reflect regionally specific theta patterns or directionality of theta interactions [19], [23] remain to be delineated. The infralimbic component of the mPFC is considered critical for the consolidation and recall of fear extinction [3], [6]. It is interesting to note, however, that inactivation or immediate post-training low frequency stimulation of the dorsal hippocampus induces difficulties in extinction recall through interaction with the prelimbic component of the mPFC, or independently of this interaction [27]. In our study theta burst stimulation involving the dorsal hippocampal CA1 and the LA may thus have affected fear extinction through these pathways. Future studies are needed to test these possibilities, as, for instance, through stimulation-induced acceleration of extinction training, a reduction in spontaneous recovery and/or renewal/reinstatement of conditioned fear following stronger conditioning protocols.

Theta oscillations are considered spatiotemporal codes for compressing the rather long time scales of behavior into the subseconds time scale required for local circuit coordination [28]. Much evidence indicates that associative processes in fear conditioning and extinction involve activity-dependent changes of synaptic efficacy, e.g. long term potentiation (LTP; for review see [29], [30]). LTP requires temporally correlated pre- and postsynaptic activity for effective induction (for review see [31]). However, in commonly used fear conditioning paradigms, the CS is typically presented for several seconds and co-terminates with a brief aversive US, usually about one second in duration. This apparent temporal incompatibility also shows at the neural level, particularly in LA, where tone responses are strongest at stimulus onset and quickly diminish to near-baseline levels [32]. As a result, it would seem that LA neurons exhibit comparatively little tone-evoked depolarization when the US actually occurs - a conclusion that seems at odds with the requirements for effective LTP. Here, theta oscillations may provide temporal windows for local circuit coordination [28] in that they bring about recurring depolarizations during which the activity of a given local cell population is coupled with that of afferent neurons, augmenting synaptic plasticity with little increase in firing rates. In fact, theta phase-related unit activity exists at the recording sites, as indicated in the present and a previous study [23], supporting the notion that phase-locked synaptic activity contributes to local theta LFP generation in these structures (Figure S2).

Such coordination of neural populations would seem important also during extinction, since this process involves a reorganization of the fear memory, rather than its erasure [33]. In fact, extinction training does not abolish CS responsiveness of all LA neurons, but rather causes a shift in their spatial distribution, in that the magnitude of CS-evoked responses declines in the dorsal [32], [34], but persists in the ventral part of the LA [34]. Similarly, in basal amygdala nuclei, one neuronal population increases CS responsiveness with extinction while another group of neurons expresses CS-evoked activity in a context-dependent manner in renewal tests [35]. More specifically, around 25% of neurons were found to maintain an increased CS-responsiveness after extinction training, and an additional 15% acquired an increased CS-responsiveness as a result of extinction training [35]. Finally, a third group of neurons, accounting for 13% of the cells, expressed CS-evoked activity in a context-dependent manner in renewal tests [35]. Different types of neurons in the amygdala thus seem to signal fear memory and extinction, shifting the balance between the expression of fear and/or extinction after conditioning. The results of the present study indicate that theta coupling may functionally connect the relevant populations of LA neurons and the infralimbic PFC during CS-evoked freezing at stages of extinction recall, much like LA-CA1 theta coupling during fear memory recall.

Additional support for this conclusion comes from studies that examined the hippocampal-dependence of conditioned fear to cues before and after extinction training. Whereas dorsal hippocampal lesions and inactivations do not block expression of conditioned fear responses [36], [37], the same manipulations performed after extinction training do [37], [38]. Indeed, dorsal hippocampal lesions and inactivations after extinction training prevent the context-dependent renewal of conditioned fear [37], [38]. The relevant neuronal populations in the dorsal hippocampal CA1 may functionally connect to the infralimbic PFC through theta coupling, thereby gating conditioned fear after extinction.

Overall, recruitment into theta oscillations of specific types of neurons, and the correlation of oscillatory activity, may thus contribute to a population code of conditoned stimuli during stages of fear memory recall before and after extinction.

## Materials and Methods

### Animals and surgical procedures

A total of 47 male C57Bl/6J mice (M&B Taconic, Berlin, Germany) were used in accordance with the regulations of German law and as approved by the Bezirksregierung Münster (AZ 50.0835.1.0, G 53/2005). Animals were kept in a 12 hour light/dark cycle, provided with food and water *ad libitum* and included in the experiment at 8 to 12 weeks of age. All surgical procedures were carried out under deep pentobarbital anesthesia (50 mg/kg i.p.). Electrodes were implanted in the left hemisphere at the following stereotactical coordinates [39]: CA1: -1.94/1/1.25 mm, LA: -2.06/3.25/3.2 mm, and mPFC: 1.75/0.3/2.0 mm from bregma. Electrodes were fixated with dental cement and for LFP and microstimulation electrodes, the external ends of the wires were fed through a rubber socket, which then was fixated with dental cement as well. All experiments involved a dedicated reference and a ground electrode, positioned close to the midline over nasal (3.5/1.0 mm from bregma) and cerebellar region (5.8/0.5 mm from bregma) of the right hemisphere for reference and ground, respectively. After experiments, animals were sacrificed with an overdose of isoflurane inhalation; brains were rapidly removed and preserved in 4% formalin. Cryosections were made and stained with cresyl violet to verify the electrode positions (Figure 2).

### Fear conditioning

After 4–6 days of surgical recovery, animals underwent fear conditioning as previously described (Figure 1A) [18], [20]. In brief, on day -2, mice were adapted to the fear conditioning apparatus (TSE, Bad Homburg, Germany) and exposed to 6 neutral tones (CS-, 2.5 kHz, 85 dB, 10 s, 20 s inter-stimulus interval (ISI)). This adaptation session was repeated once. Fear conditioning took place on the following two days (day -1 and day 0) and consisted of 3 tones (CS+, 10 kHz, 85 dB, 10 s, ISI randomized 10–30 s), co-terminating with a 1 s foot shock (scrambled, 0.4 mA). Twenty-four hours later, extinction training was started (day 1). Under slight Forene anesthesia (isofluran, 1-chloro-2,2,2-trifluoroethyl- difluoromethylether) animals were connected to a swivel commutator of the recording device and after a recovery period of 30 min the experiment started. The protocol involved 6 retrieval sessions (R1-R6, 6 min each, separated by 30 mins), in which both the CS- and CS+ were presented (4 times each) in a neutral context. Animals remained connected between the different extinction training sessions. Retention of extinction learning was then assessed on the following day (day 2) through two sessions in the extinction context (E1, E2; identical to the retrieval sessions).

### Behavioral analyses

Behavioral expressions were evaluated online by an experienced experimenter and analyzed offline. Freezing, immobility except for respiratory movements, was taken as a behavioral measurement of fear. In each session (R1–R6, E1-2) freezing time was calculated as percentage during the first CS- and CS+ presentations. Furthermore, additionally expressed behaviors were monitored, for example, risk-assessment (alert observing and stretched attending), rearing and exploration.

### Electrophysiology

For recordings of LFPs (13 animals) custom-made steel electrodes were employed (Franco Corradi, Italy). For recordings of unit activity related to LFPs (8 animals), eight-channel electrode bundles (insulated PI microwire; Plexon Inc., Dallas, Texas, USA) were used. Local microstimulation experiments (26 animals) were carried out using bipolar steel electrodes made from double-stranded wires (Franco Corradi, Italy).

For LFP experiments, recorded waveforms were fed through a differential amplifier (Science Products DPA-2F), band-pass filtered from 1 to 30 Hz, and stored on a personal computer via an A/D interface (CED Power 1401, sampling rate: 1 kHz). An automated artifact detection routine assessed the LFP variance in a sliding 300 ms window and data segments exceeding a threshold were excluded from further analysis (individual thresholds were set for each animal and kept constant for the processing of all sessions). All data segments surviving this routine were shuffled, rendering order, animal, session, behavior, and CS unknown to the experimenter, and then visually inspected and rejected if found to contain further movement artifacts.

For simultaneous recordings of unit and LFP activity, microwire sets were attached to a unity-gain headstage connecting to a preamplifier. Data were bandpass filtered between 0.7 and 154 Hz and 100 Hz and 13 kHz for LFPs and unit recordings, respectively, and processed using the Multi Acquisition Processor (MAP) system (Plexon Inc.) in single-channel mode (sampling rate 1 kHz for LFPs and 40 kHz for unit activity) for real-time threshold setting and waveform discrimination. Spike waveforms with a signal-to-noise ratio ≥2 were considered for additional off-line analysis using three-dimensional plots of principal component scores (ValleySeeking, Offline Sorter; Plexon Inc.). Noise was determined as the background level of activity over a time period of 10 seconds (CS+). Timestamps of neural spiking and field potential recordings were exported to NeuroExplorer (NEX Technologies) for further analysis.

For local electrical microstimulation, bipolar steel electrodes were implanted in CA1 and LA of the left hemisphere (coordinates as above). Stimulus trains were delivered five seconds after each CS+ presentation in each retrieval session (R1-R6), consisting of 50 theta bursts (each burst composed of 5 square pulse stimuli at 200 Hz, each 0.1 ms in duration, 0.1 mA; inter-burst interval 200 ms). Stimuli at CA1 and LA were applied at the same phase (“in phase”) or at 180° phase offset (“anti phase”). Retention of extinction (E1, E2) was performed on the next day without electrical microstimulation.

### Histology

At the end of the electrophysiological experiments, positions of the electrode tips were marked through an electrolytic lesion (anodal current 0.4 mA, 2 s), animals were sacrificed with an overdose of pentobarbital (100 mg/kg), and probe locations were histologically verified by cresyl violet staining in 50 µm brain slices (Figure 2) in comparison to “The Mouse Brain in Stereotaxic Coordinates” [39].

### Cross-Correlations

Cross-correlograms from all three combinations of channels (CA1-LA; CA1-mPFC; LA-mPFC) were computed using Matlab's xcorr function with coefficient normalization (‘coeff’ – option), which normalizes the sequence so the autocorrelations at zero lag are identically 1.0 for both signals. This avoids possible amplitude-related bias. In all cross-correlation analyses, the middle peak at zero latency was discarded as it captures non-oscillatory linear relations as well as oscillatory interactions and the former were not of interest in this study. Middle peaks were present in almost all cross-correlograms computed and usually fell within the range of ±3 ms. Such zero-latency coupling can be caused by volume conduction [16] and discarding the middle peak thus avoids contamination by the passive spread of current through brain tissue. The magnitude value of the most central negative peaks (corresponding to latencies of ±π) and the most central positive peaks (corresponding to latencies of ±2π) were taken as a measure of purely oscillatory interaction between the channels (Figure 4A) and statistically assessed via ANOVA as described in the text.

## Supporting Information

Figure S1 **Theta power throughout the experiment.** (A) Theta power in response to presentation of the CS+ for all recording sites and all sessions. Each horizontal line represents a data segment from one animal during the respective session. Dark blue horizontal lines represent data segments that did not survive artifact rejection and were therefore not included in the analysis. The white trace depicts the average power spectrum for the given channel and session. Note that considerable theta power is observed in all regions throughout the entire experiment. (B) Theta power in response to presentation of the CS-. Note that, as for the CS+, CS- presentation was accompanied by considerable theta activity throughout the course of the experiment.
(TIF)

Figure S2 **Simultaneously recorded LFP and unit activity in CA1 (upper row), LA (middle row), and mPFC (bottow row).** (A) CS+ - related activity. Peri-stimulus raster of activity in identified units, calculated 10 seconds before and during CS+ presentation (stimulus onset at time zero). Histograms are averages of activity during 4 consecutive CS+ presentations in retrieval session 1 (individually displayed in horizontal spike trains). (B) Theta phase-related unit activity during R1. Shown are original LFP waveforms (low pass filtered at 12 Hz; upper traces), and simultaneously recorded unit activity (bottom traces). (C) Average phase distribution of all identified units with significant theta-phase locking during R1. Note that the preferred phase predominantly occurred in the trough of the oscillation (around 180°) in CA1 and LA, and at the peak of the oscillation (around 0°) in mPFC. Phase distributions were analyzed in identified units as follows. Theta peaks were identified in LFP waveforms, and time span between peaks was normalized to 2π. Phase relation of concomitant unit activity was assessed using Rayleigh's test for uniformity, and the phase distribution was plotted for each unit in circular coordinates with 10° degree bin width. A total of 28 neurons were analyzed in CA1, 20 in LA and 36 in mPFC, of which 18%, 15%, and 17%, respectively, displayed preferential theta phase locking. Phase distributions of unit activity with preferred phase were averaged in each of the three regions, values were normalized, plotted and values exceeding 0.8 highlighted.
(TIF)

Figure S3 **Effects of CA1-LA microstimulation “in-phase” on electrical activity in LA and CA1 (A), on correlation between LA and CA1 (B) and on freezing in non fear-conditioned controls (C).** (A) Representative time frequency spectrograms and cross-correlograms of CA1 and LA activity at baseline before (20 s prior to the first CS- presentation) and following microstimulation after CS+ presentation (5 s period after microstimulation) at R1, and R6, and after CS+ presentation at E1 on the next day. Note the increase in correlated theta activity in CA1 and LA post stimulation at R1 and R6. Characters indicate animal behavior (f: freezing; w: risk-assessment). (B) Averages of cross-correlation values between LA and CA1 at baseline, post-stimulation R1, and R6 and post CS+ presentation at E1. Note the stimulation induced increase of correlation between CA1 and LA and the decrease at E1. (One-way ANOVA (p<0.05) followed by Tukey's post hoc test) (C) Freezing behavior in non fear conditioned mice upon CS presentation in the routine training program, with (CS+) and without (CS-) electrical microstimulation “in phase” of CA1 and LA during R1-R6. Note the low level of freezing throughout sessions, and the lack of influence of microstimulation. Statistical comparison of CS+ and CS- revealed no significant differences (ANOVA with repeated measurements). Values are mean ± SEM from recordings in 5 mice.
(TIF)
