# Supplementary figures and images for: Patterns of Coupled Theta Activity in Amygdala-Hippocampal-Prefrontal Cortical Circuits during Fear Extinction
Source: PLoS One. 2011 Jun 28;6(6):e21714. doi: 10.1371/journal.pone.0021714 (PMC3125298; doi:10.1371/journal.pone.0021714)

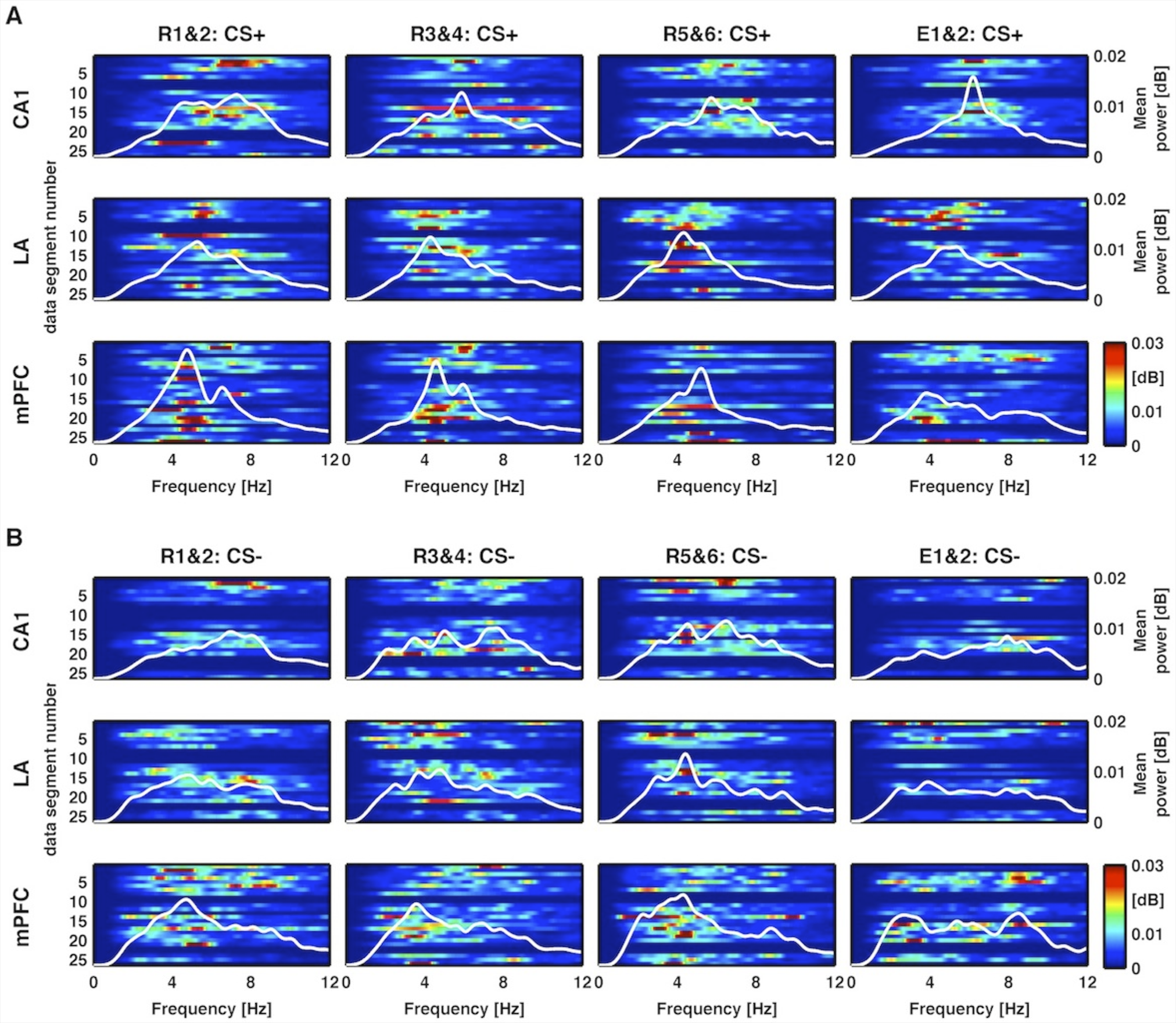

Supplement: Figure S1 — Theta power throughout the experiment. (A) Theta power in response to presentation of the CS+ for all recording sites and all sessions. Each horizontal line represents a data segment from one animal during the respective session. Dark blue horizontal lines represent data segments that did not survive artifact rejection and were therefore not included in the analysis. The white trace depicts the average power spectrum for the given channel and session. Note that considerable theta power is observed in all regions throughout the entire experiment. (B) Theta power in response to presentation of the CS-. Note that, as for the CS+, CS- presentation was accompanied by considerable theta activity throughout the course of the experiment. (TIF) [file pone.0021714.s001.tif]

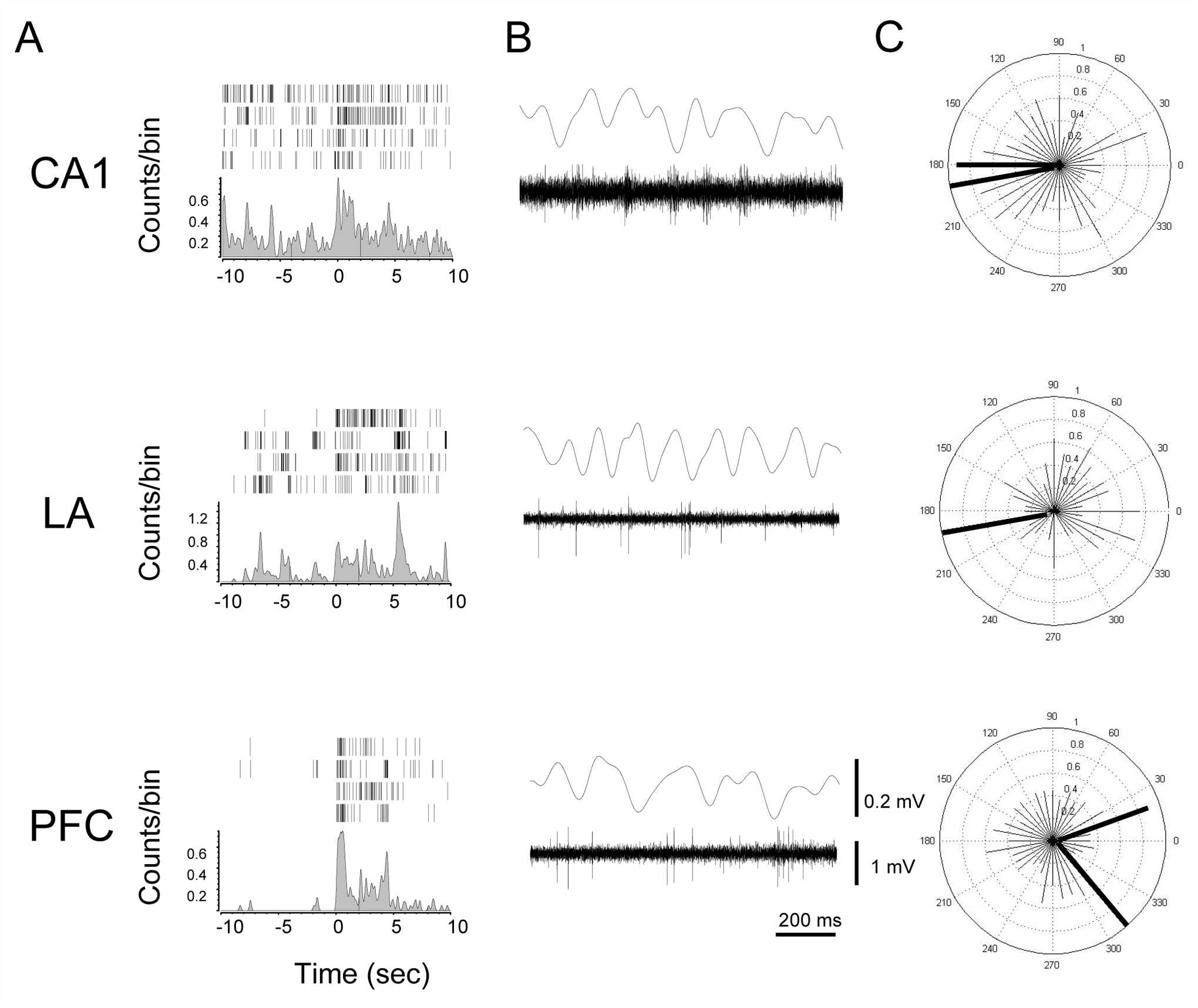

Supplement: Figure S2 — Simultaneously recorded LFP and unit activity in CA1 (upper row), LA (middle row), and mPFC (bottow row). (A) CS+ - related activity. Peri-stimulus raster of activity in identified units, calculated 10 seconds before and during CS+ presentation (stimulus onset at time zero). Histograms are averages of activity during 4 consecutive CS+ presentations in retrieval session 1 (individually displayed in horizontal spike trains). (B) Theta phase-related unit activity during R1. Shown are original LFP waveforms (low pass filtered at 12 Hz; upper traces), and simultaneously recorded unit activity (bottom traces). (C) Average phase distribution of all identified units with significant theta-phase locking during R1. Note that the preferred phase predominantly occurred in the trough of the oscillation (around 180°) in CA1 and LA, and at the peak of the oscillation (around 0°) in mPFC. Phase distributions were analyzed in identified units as follows. Theta peaks were identified in LFP waveforms, and time span between peaks was normalized to 2π. Phase relation of concomitant unit activity was assessed using Rayleigh's test for uniformity, and the phase distribution was plotted for each unit in circular coordinates with 10° degree bin width. A total of 28 neurons were analyzed in CA1, 20 in LA and 36 in mPFC, of which 18%, 15%, and 17%, respectively, displayed preferential theta phase locking. Phase distributions of unit activity with preferred phase were averaged in each of the three regions, values were normalized, plotted and values exceeding 0.8 highlighted. (TIF) [file pone.0021714.s002.tif]

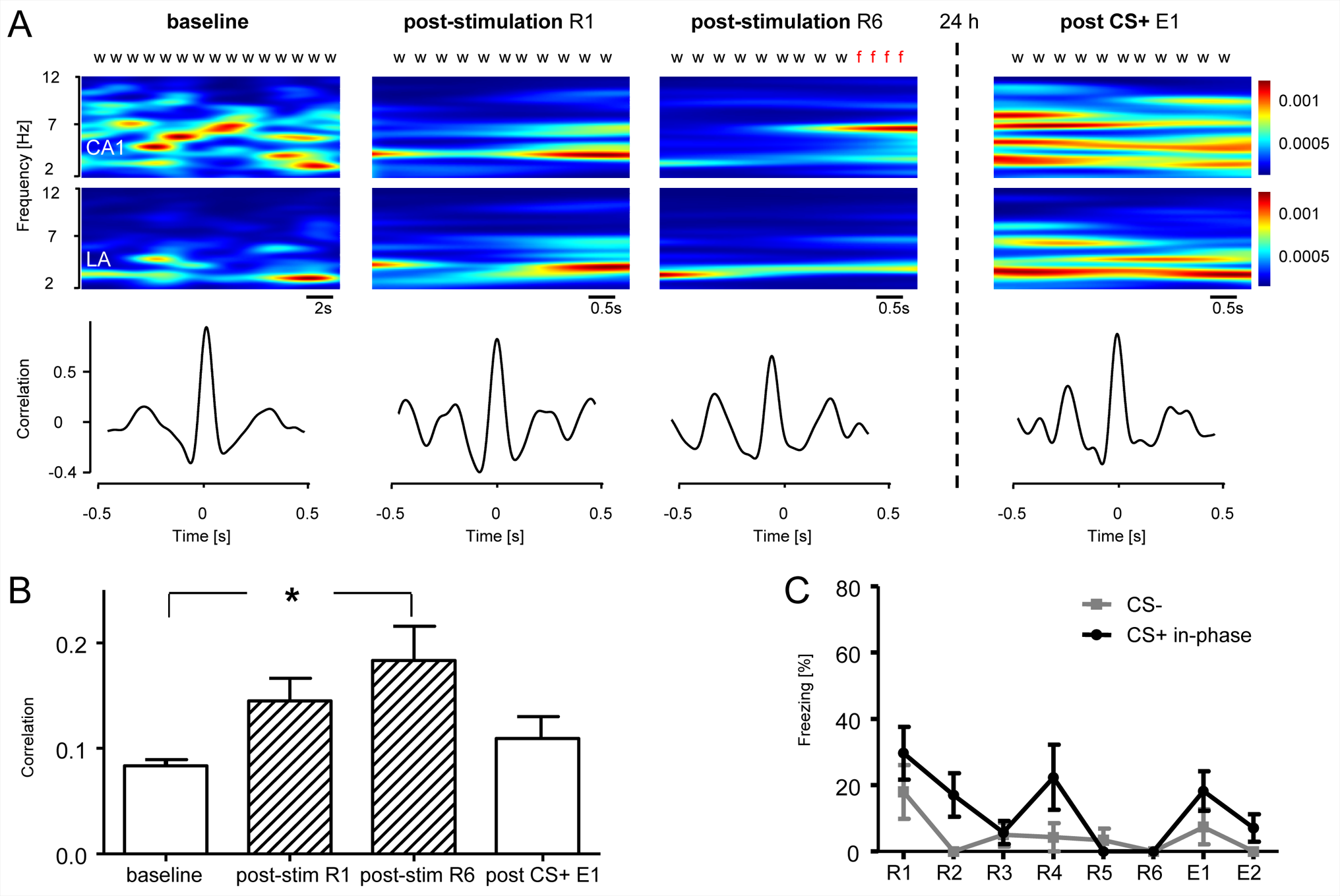

Supplement: Figure S3 — Effects of CA1-LA microstimulation “in-phase” on electrical activity in LA and CA1 (A), on correlation between LA and CA1 (B) and on freezing in non fear-conditioned controls (C). (A) Representative time frequency spectrograms and cross-correlograms of CA1 and LA activity at baseline before (20 s prior to the first CS- presentation) and following microstimulation after CS+ presentation (5 s period after microstimulation) at R1, and R6, and after CS+ presentation at E1 on the next day. Note the increase in correlated theta activity in CA1 and LA post stimulation at R1 and R6. Characters indicate animal behavior (f: freezing; w: risk-assessment). (B) Averages of cross-correlation values between LA and CA1 at baseline, post-stimulation R1, and R6 and post CS+ presentation at E1. Note the stimulation induced increase of correlation between CA1 and LA and the decrease at E1. (One-way ANOVA (p<0.05) followed by Tukey's post hoc test) (C) Freezing behavior in non fear conditioned mice upon CS presentation in the routine training program, with (CS+) and without (CS-) electrical microstimulation “in phase” of CA1 and LA during R1-R6. Note the low level of freezing throughout sessions, and the lack of influence of microstimulation. Statistical comparison of CS+ and CS- revealed no significant differences (ANOVA with repeated measurements). Values are mean ± SEM from recordings in 5 mice. (TIF) [file pone.0021714.s003.tif]
